# Extracellular Self-DNA (esDNA), but Not Heterologous Plant or Insect DNA (etDNA), Induces Plasma Membrane Depolarization and Calcium Signaling in Lima Bean (*Phaseolus lunatus*) and Maize (*Zea mays*)

**DOI:** 10.3390/ijms17101659

**Published:** 2016-09-29

**Authors:** Francesca Barbero, Michela Guglielmotto, Andrea Capuzzo, Massimo E. Maffei

**Affiliations:** 1 Department of Life Sciences and Systems Biology, University of Turin, Via Quarello 15/a, 10135 Turin, Italy; francesca.barbero@unito.it; 2 Neuroscience Institute of Cavalieri Ottolenghi Foundation (NICO), University of Turin, Regione Gonzole 10, Orbassano, 10043 Turin, Italy; michela.guglielmotto@unito.it; 3 Biosfered Srl, Academic Spin-Off of the University of Turin, Via Quarello 15/a, 10135 Turin, Italy; a.capuzzo@biosfered.com

**Keywords:** self-DNA recognition, plasma membrane potential, calcium signaling, Lima bean, maize, *Spodoptera littoralis*

## Abstract

Extracellular self-DNA (esDNA) is produced during cell and tissue damage or degradation and has been shown to induce significant responses in several organisms, including plants. While the inhibitory effects of esDNA have been shown in conspecific individuals, little is known on the early events involved upon plant esDNA perception. We used electrophysiology and confocal laser scanning microscopy calcium localization to evaluate the plasma membrane potential (Vm) variations and the intracellular calcium fluxes, respectively, in Lima bean (*Phaseolus lunatus*) and maize (*Zea mays*) plants exposed to esDNA and extracellular heterologous DNA (etDNA) and to etDNA from *Spodoptera littoralis* larvae and oral secretions. In both species, esDNA induced a significant Vm depolarization and an increased flux of calcium, whereas etDNA was unable to exert any of these early signaling events. These findings confirm the specificity of esDNA to induce plant cell responses and to trigger early signaling events that eventually lead to plant response to damage.

## 1. Introduction

The degradation of plant tissues releases organic matter composed of different classes of compounds, including DNA. This event occurs during natural decomposing events, such as plant littering, or during cell disruption and degradation caused by cell apoptosis, herbivores, and pathogens. When damaged plant cells release DNA it can be further metabolized to produce DNA fragments in a variable size range (usually between 50 and 2000 bp (base pair)). It has been recently argued that this extracellular fragmented self-DNA (esDNA) acts as a signaling molecule (or second messenger) able to trigger inhibitory effects on conspecific plants [1,2,3]. Moreover, plants use this esDNA to build resistance against pathogens and as a means of maintaining biodiversity [4]. Although it is not fully understood whether esDNA is generated either through the degradative action of microorganisms or herbivores’ digesting system or released actively by the living plant cells, the current evidence indicates that esDNA could function as a conspecific stress signaling molecule acting in damage-associated molecular patterns (DAMPs) [5].

Although not directly measured, indirect evidence shows that application of common bean esDNA to common bean leaves induces reactive oxygen species (ROS) development and triggers resistance-related mechanisms, which are not elicited by extracellular heterologous DNA (etDNA) [6]. These responses are similar to plant responses to biotic attack, where ROS production, along with calcium signaling and plasma membrane potential (Vm) depolarization are among the early signaling events preceding the buildup of chemical defense [7,8,9]. In plant–biotroph interactions, the plasma membrane is the first site for the reception of external molecules were elicitor–receptor reactions produce variations in the Vm, which is defined as the difference in the electrochemical gradient between the interior and exterior of the plant cell [10]. In this kind of interaction, Vm variations often depend on the calcium-dependent opening of inward potassium channels, which eventually reduce the Vm to a depolarized state [11]. These events precede gene expression and the production of plant responses [12,13]. Therefore, it would be interesting to assess whether esDNA is able to induce similar responses in plants.

So far, no esDNA receptor has been identified in plant cells, whereas in animal cells self-DNA released from apoptotic cells triggers the innate immune activation and mediates immune response [14]. In animal cells, different DNA pattern recognition receptors and sensors have been identified. These include among others cyclic GMP–AMP (cGAMP) synthase (cGAS) [15], DNA-sensing inflammasome receptor absent in melanoma 2 (AIM2) [16], cationic antimicrobial peptide LL37 [17], and self-DNA bound to Toll-like receptor 9 (TLR9) [18,19,20].

In order to evaluate the signaling pathways involved in plant esDNA perception, we extracted and fragmented DNA from Lima bean, maize, and from the larvae and the oral secretions of the generalist herbivore *Spodoptera littoralis*. Here we show that, in both species, only esDNA is able to induce a significant Vm depolarization and calcium signaling, and that etDNA from both plants and the herbivore exert no effects on these early events.

## 2. Results

### 2.1. DNA Sonication and DNA Fragment Analysis

**Figure 1:**
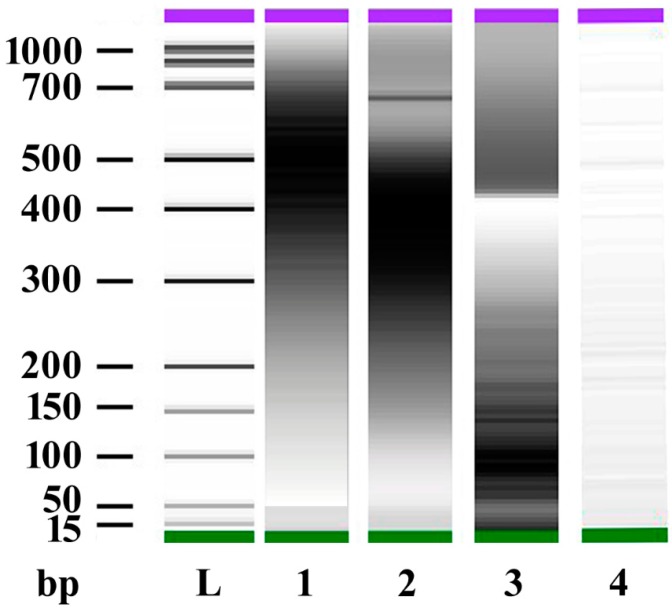
Bioanalyzer capillary gel electrophoresis densitometry plot from electropherograms of DNA fragments obtained by sonication of DNA extracts from *Zea mays* (lane 1), *Phaseolus lunatus* (lane 2), *Spodoptera littoralis* (lane 3), and *S. littoralis* oral secretions (lane 4). See Materials and Methods for more details. *Z. mays* and *P. lunatus* fragmented DNA are in the range between 300 and 700 bp. *S. littoralis* fragmented DNA is mainly composed of fragments between 15 and 300 bp, whereas *S. littoralis* oral secretions fragmented DNA shows small amounts of fragments throughout the bp range (this image has been digitally enhanced to visualize the bands). L, base pair ladder lane; bp, base pair size reference bars.

In order to evaluate Lima bean and maize responses to esDNA and etDNA, we extracted the total DNA from both plants as well as from the oral secretions (OS) and the larvae of the generalist herbivore *S. littoralis*. Sonication of DNA (see Section 4.2) yielded fragments of different bp, which were analyzed by capillary gel electrophoresis (CGE, Agilent 2100 Bioanalyzer, Agilent Technologies, Santa Clara, CA, USA). Figure 1 shows the gel-like representation (densitometry plot) of the electropherograms from analyzed samples. Sonicated DNA from maize and Lima bean produced fragments in the range between 300 and 700 bp. Sonicated DNA from *S. littoralis* was mainly composed of fragments between 15 and 300 bp, whereas sonication of *S. littoralis* OS DNA showed small amounts of fragments between 15 and 1000 bp.

### 2.2. Extracellular Self-DNA (esDNA) Induces Plasma Membrane Potential (Vm) Depolarization

**Figure 2:**
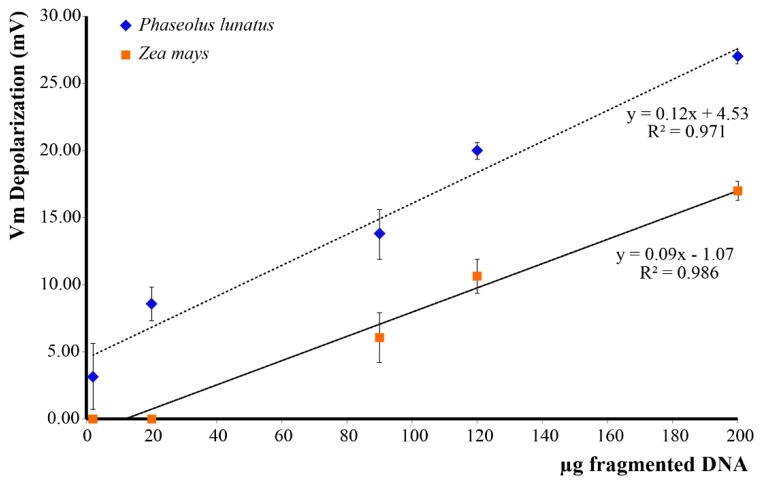
Plasma membrane potential (Vm) depolarization caused by increasing concentrations of extracellular self-DNA (esDNA) in Lima bean and maize. Error bars represent standard error (*n* = 8–10). Regression equation and the coefficient of determination are indicated for each species.

In order to assess the effect of esDNA on Lima bean and maize, we evaluated the Vm depolarization values as a function of esDNA concentration. We found that Lima bean leaves, which normally possess a higher Vm (ca. 140–150 mV), responded to esDNA with a higher Vm depolarization with respect to maize leaves. The minimal esDNA concentration able to exert a Vm depolarization was <2 µg·mL^−1^ in Lima bean, whereas in maize the esDNA minimal concentration was 12 µg·mL^−1^ (Figure 2). A linear response was found for both plants up to 200 µg·mL^−1^ esDNA.

**Figure 3:**
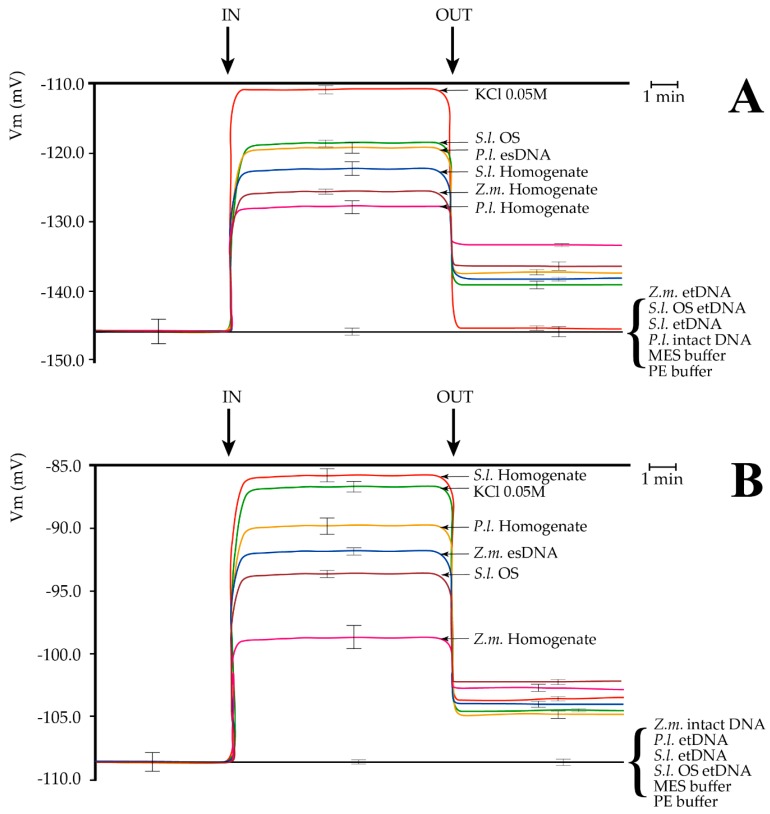
Plasma membrane potential (Vm) depolarization caused by different treatments in Lima bean (*Phaseolus lunatus*) and maize (*Zea mays*) leaves. (**A**) Perfusion of Lima bean leaves with lima bean esDNA caused a Vm depolarization similar to *S. littoralis* oral secretions (OS). Leaf and larval homogenates were also found to induce a significant Vm depolarization. No effect on Vm was found after buffers, *P.l.* intact DNA, or extracellular heterologous DNA (etDNA) application; (**B**) Perfusion of maize leaves with maize esDNA caused a Vm depolarization similar to *S. littoralis* OS. Leaf and larval homogenates were also found to induce a significant Vm depolarization. No effect on Vm was found after buffers, *Z.m.* intact DNA or etDNA application. *P.l.*, *Phaseolus lunatus*; *Z.m*., *Zea mays*; *S.l*., *Spodoptera littoralis*; OS, oral secretions. IN, time of perfusion of different solutions; OUT, time of washing with fresh buffer. A time scale is indicated. Error bars represent standard error (*n* = 8–10).

Having assessed the dose–response effect of esDNA, we compared the response of esDNA and etDNA with other factors that are able to induce a Vm depolarization (Figure 3).

In Lima bean, perfusion with a 50 mM KCl induced a typical Vm depolarization [21]. A lower Vm depolarization occurred when the *S. littoralis* OS were assayed, as reported for this species [11,22]. Administration of 200 µg·mL^−1^ esDNA prompted a significant Vm depolarization, with Vm responses similar to *S. littoralis* OS. We also compared leaf homogenates of Lima bean and maize as well as *S. littoralis* larval homogenate. The highest Vm depolarization was found for *S. littoralis* larval homogenate, followed by maize and Lima bean homogenates, respectively (Figure 3A). In all experiments, washing out the solutions with a fresh buffer reduced Vm depolarization, but never to the initials values (with the sole exception for KCl in Lima bean). Perfusion of either maize or *S. littoralis* larvae or 200 µg·mL^−1^ OS etDNA did not cause any significant Vm depolarization. Same results were found when 200 µg·mL^−1^ intact and unfragmented Lima bean DNA, MES (4-Morpholineethanesulfonic acid), and PE (5 mM Tris/HCl, pH 8.5) buffers were assayed (Figure 3A).

In order to evaluate whether the specificity of esDNA was only present in Lima bean, we perfused maize leaves with the same solutions used for Lima bean. In maize, the highest Vm depolarization was found after *S. littoralis* homogenate perfusion, which prompted the same Vm depolarization as 50 mM KCL. Lima bean homogenate caused a lower Vm depolarization and was followed by maize esDNA Vm depolarization. *S. littoralis* OS also induced a significant Vm depolarization in maize leaves, although with a lower effect with respect to Lima bean. A small but significant Vm depolarization was induced by perfusing maize leaves with maize leaf homogenate (Figure 3B). As found for Lima bean, washing out the solutions with fresh buffer reduced Vm depolarization, which remained significantly higher than the initial values. Perfusion with intact and unfragmented maize DNA, and etDNA from either Lima bean, *S. littoralis* larvae, *S. littoralis* OS, or MES and PE buffers did not cause any significant change to the maize leaf Vm (Figure 3B).

### 2.3. Cation Capillary Electrophoresis of Leaf and Larvae Homogenates and of Fragmented DNA

In order to understand the reasons underlying the Vm depolarization found after perfusion with the leaf and larval homogenates, and in order to assess the contribution of cations to the observed Vm variations, we extracted and analyzed the cation content by capillary electrophoresis.

Table 1 shows the cation composition of the two buffers used to prepare the solutions, the three homogenates, and the cation analysis of the sonicated DNA from maize and Lima bean. The results show that most of the Vm depolarization of the three homogenates is mainly associated with the high content of potassium, whereas the content of this cation of both sonicated DNAs is not significantly different (*p* > 0.05) from the cation content in the PE buffer. These results, along with the absence of Vm variations upon PE buffer perfusion, indicate that esDNA-induced Vm depolarization does not depend on cation content, because etDNA is unable to induce a Vm depolarization.

### 2.4. esDNA Induces Calcium Signaling

**Figure 4:**
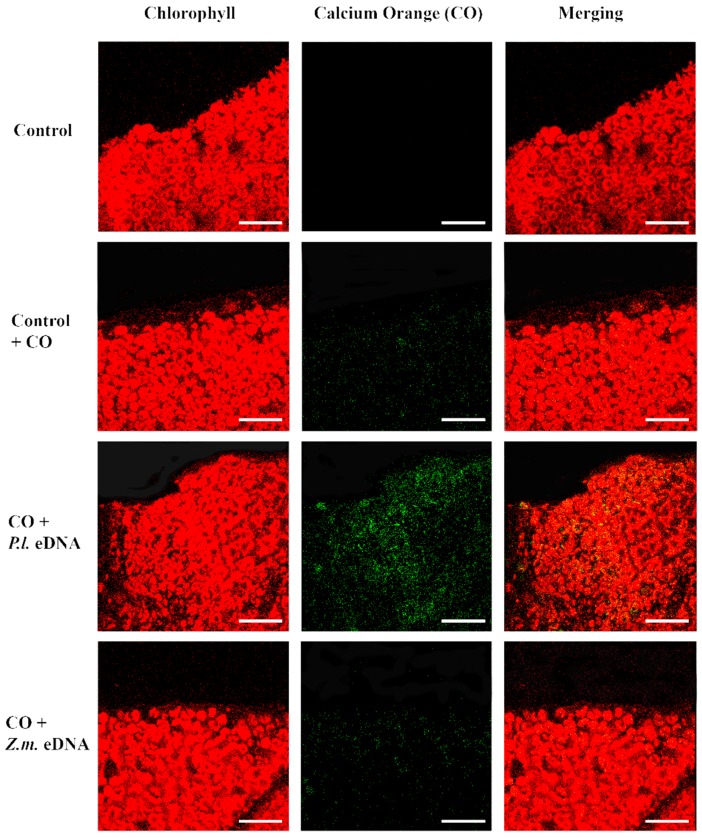
Intracellular calcium variations in Lima bean leaves upon different treatments. False-color image analysis reconstructions from confocal laser-scanning microscope observations, and fluorochemical intracellular Ca^2+^ localization. Fifty microliters of 200 µg·mL^−1^ of either esDNA or extracellular heterologous DNA (etDNA) were applied, and after 30 min the calcium signature was observed. Pictures represent portions of the Lima bean leaf blade where the green fluorescence refers to binding of calcium orange with Ca^2+^, whereas the chloroplasts are evidenced by a bright red color caused by chlorophyll fluorescence. Scale bar (100 µm) is indicated on the figures. CO, calcium orange; *P.l.*, *Phaseolus lunatus*; *Z.m*., *Zea mays*.

In plant–herbivore interactions, Vm depolarization is associated with the release of cytosolic calcium [Ca^2+^]_c_ from the internal stores [23,24,25,26]. Confocal laser scanning microscopy allows the localization and the semiquantitative evaluation of [Ca^2+^]_c_ by using selective calcium indicators [27]. We used calcium orange to evaluate the response of Lima bean leaves to application of esDNA and maize etDNA. A preliminary dose-dependent analysis was performed, and we found that 200 µg·mL^−1^ esDNA induced a significant response in both plant species (data not shown). Figure 4 shows the chlorophyll and calcium orange fluorescence and the merging of the two signals in controls (where no treatment is done), in leaves treated with the sole calcium orange (in order to assess the minimum calcium response) and after perfusion with 50 µL of 200 µg·mL^−1^ esDNA and maize etDNA. The images show clearly that esDNA induces a strong fluorescence associated with the [Ca^2+^]_c_ efflux, whereas the signal observed after maize etDNA has a similar fluorescence as the calcium orange control (Figure 4).

**Figure 5:**
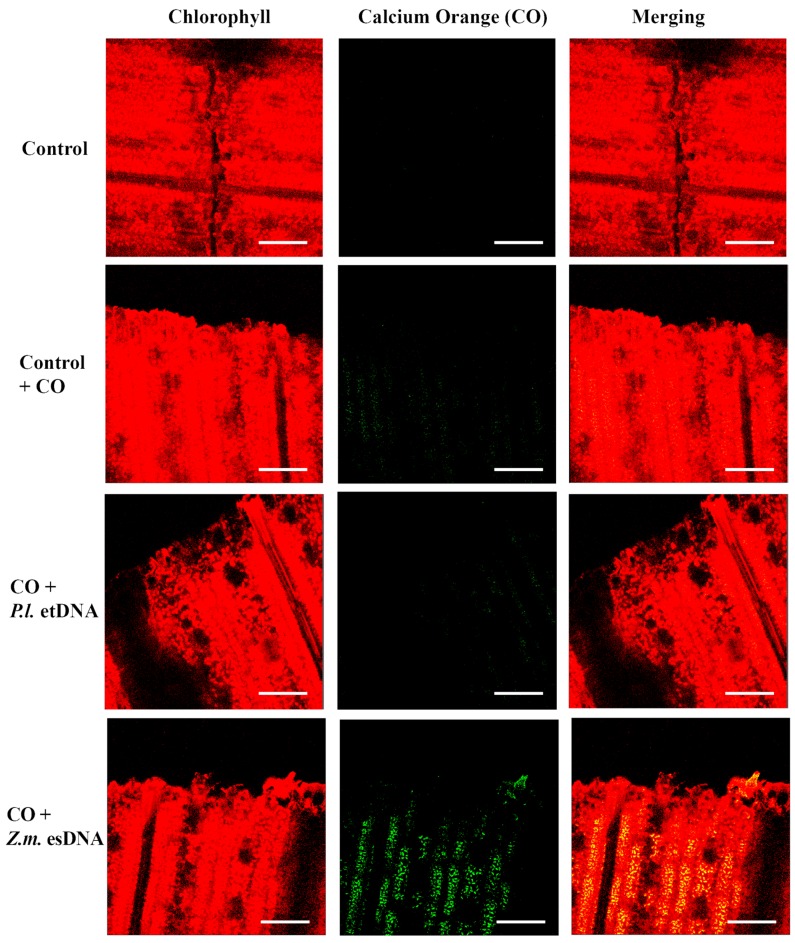
Intracellular calcium variations in maize leaves upon different treatments. False-color image analysis reconstructions from confocal laser-scanning microscope observations, and fluorochemical intracellular Ca^2+^ localization. Fifty microliters of 200 µg·mL^−1^ of either esDNA or etDNA were applied, and after 30 min the calcium signature was observed. Pictures represent portions of the maize leaf blade where the green fluorescence refers to binding of calcium orange with Ca^2+^, whereas the chloroplasts are evidenced by a bright red color caused by chlorophyll fluorescence. Scale bar (100 µm) is indicated on the figures. CO, calcium orange; *P.l.*, *Phaseolus lunatus*; *Z.m*., *Zea mays*.

The fluorimetric localization of [Ca^2+^]_c_ in maize leaves showed similar results as observed in Lima bean (Figure 5). Even in this case, 50 µL of 200 µg·mL^−1^ esDNA prompted a higher fluorescence signal with respect to that observed in controls with calcium orange. In both plant species, esDNA prompted a sustained calcium elevation.

**Figure 6:**
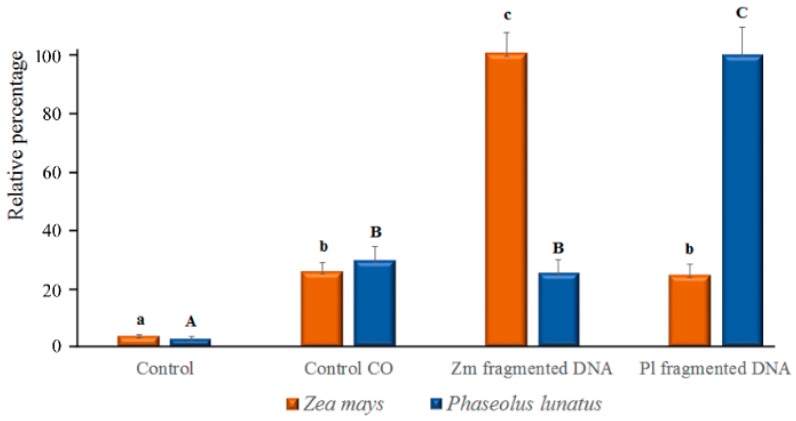
Relative percentage of the Ca^2+^ release shown in confocal Figure 4 and Figure 5. In both species, a significant (*p* < 0.05) difference was found between controls and fragmented DNA only when esDNA was used. Error bars represent standard error (*n* = 8–10). Different letters (small caps for maize and capital letters for Lima bean) indicate significant differences (*p* < 0.05, Tukey–Kramer HSD) between treatments and controls.

Image analysis of calcium orange fluorescence confirmed the increased trend of [Ca^2+^]_c_ in esDNA-treated leaves (Figure 6). In both Lima bean and maize, only a faint fluorescence was found after perfusion with etDNA; however, image analysis (see also Section 4.4) indicated that no significant differences in [Ca^2+^]_c_ were found between etDNA treatments and controls (Figure 6).

## 3. Discussion

Recognition of self is one of the main strategies that organisms adopt to react quickly to cell and tissue injuries. Endogenous signals are the first effectors of a rapid response to external damage and their nature varies from single ions, such as calcium, to complex molecules, including macromolecules like DNA. In plants, just like in animal cells, DAMPs released upon cell damage trigger cascades of events, eventually leading to a coordinated response [6]. In animal cells, several DAMPs receptors/sensors have been identified, including those able to sense cytosolic DNA [28,29]. The recent finding that plant esDNA acts as an inhibitor of growth and development [1] triggered a series of empirical questions, as recently pointed out by Martin Heil and coworkers [4,5].

In this work, we showed that plant esDNA is specific and able to trigger early events associated to the perception and transduction of a signal, such as the plasma membrane potential alteration and the cytosolic influx of calcium ions. By applying conspecific and heterologous sonicated DNA we showed that Lima bean and maize respond actively to esDNA, whereas their perceiving system is unable to detect etDNA. We found that the wounded tissue is able to perceive the esDNA signal and that this signal is able to spread to distant cells (as shown both in Vm measurements and especially in confocal calcium imaging). This response, which is typical of tissues responding to herbivory [13,30,31] and that depends on the symplastic connections of plants cells [11], indicates that the same sensing system might apply for both herbivory and esDNA perception.

In order to separate direct from indirect effects, we compared the response of plant cells to insect OS, insect and plant homogenates, and integral DNA. As expected, *S. littoralis* OS, which contains oligosaccharide elicitors [11], triggered a Vm depolarization both in Lima bean and in maize, which was similar to esDNA application. However, OS etDNA was unable to induce any response in both plants, thus indicating that the Vm depolarization was exclusively depending on reception of the insect oligosaccharidic elicitor [30]. It is known that plant homogenates may trigger plant response when applied exogenously [6]. It is quite probable that these homogenates contain, among others, DNA molecules. The Vm depolarization occurring upon plant homogenate treatment was revealed to be mostly associated to their high cation content, as revealed by our CE analyses, and was comparable to KCl-induced Vm depolarization. When we tested self-integral DNA, we found no responses, indicating the response solely depended on DNA fragmentation. These data are in agreement with previous work by Mazzoleni and coworkers [1,2].

Our electrophysiological dose-dependent assays revealed that concentrations as low as 2–20 µg·mL^−1^ esDNA were able to induce a significant Vm depolarization, thus justifying the hypothesis that esDNA may be realistically involved in signaling during cell and tissue damage and disruption. It is interesting to note that, despite a general common trend, the two species react with different Vm depolarization and calcium signaling to esDNA. Lima bean, a C_3_ angiosperm dicot, has a typical leaf anatomy characterized by a spongy mesophyll and a palisade layer. Previous work demonstrated that this mesophyll structure shows cells with different Vm values according to the cell type [22]. On the other hand, maize is a C_4_ angiosperm monocot, and has an internal ring of bundle sheath cells surrounded by homogeneous mesophyll cells [32]. It is possible that the different photosynthetic metabolism (C_3_ vs. C_4_) and the dimorphic nature of Lima bean mesophyll cells (with respect to the homogeneous mesophyll cells of maize) might play a significant role in plant response to esDNA both in Vm changes and in calcium signaling. Once the esDNA receptor is identified, it would be interesting to evaluate its distribution and function in dicots and monocots such as Lima bean and maize. In most of our Vm experiments, Vm values did not recover the initial value after washing the system with fresh buffer. This effect has been already observed in other plant species like tomato [9], *Ginkgo biloba* [33], and *Arabidopsis thaliana* [34]. Since changes in the Vm imply changes in the flux of ions across the plasma membrane, this observation suggests that the interaction with putative membrane receptors might not be fully reversible and that some ion channels might remain open even after the removal of the molecule.

One of the key questions relates to the nature of the response to esDNA. The specificity of esDNA vs. etDNA found in Lima bean and maize underlines the hypothetical presence of specific receptors. The rapid Vm depolarization and the significant Ca^2+^ influx observed only after esDNA imply the presence of triggering events at the plasma membrane involving activation of channels [7,8,9,11,13] and do not exclude a cascade of calcium-dependent events [26,35]. In plant–insect interactions, herbivore–induced Vm depolarization depends on variations in the K^+^ homeostasis, which is triggered by the opening of inward-rectified calcium-dependent K^+^ channels [11]. Our results suggest a similar mechanism, and further studies are underway to better assess the role of both Ca^2+^ and K^+^ channels in response to esDNA.

In plants, guard receptors detect virulence factors produced by pathogens and can be activated by a mechanism that is remarkably similar to that of mammalian Toll-like receptor 4 [36,37], whereas flagellin perception is mediated by Flagellin Sensing2 receptor (FLS2), which shares a high homology with the TLR family [38]. Emerging evidence indicates that signal transduction pathways mediated by TLR lead to calcium fluxes within cells through calcium channel activity from calcium stores [14,39]. Therefore, the observed calcium signaling upon esDNA treatment involved in plant early perception of esDNA might be compelling evidence of the presence of a receptor system. Moreover, calcium homeostasis and regulation are fundamental in plant membrane transport regulation and responses to external stimuli [40].

## 4. Materials and Methods

### 4.1. Plant and Animal Material

Lima bean plants, *Phaseolus lunatus* L. (cv. Ferry Morse var. Jackson Wonder Bush) and maize, *Zea mays* L. (cv. rostrato), were grown in a growth chamber at 23 °C and 60% humidity using daylight fluorescent tubes at approximately 170 µE·m^−2^·s^−1^ with a 14 h day/10 h night photoperiod. For Lima bean, experiments were conducted with 12–20 day-old seedlings showing two fully expanded primary leaves, which were found to be the most responsive leaves in this plant developmental stage [7]. For maize, adult nonsenescing leaves were assayed.

Larvae of *Spodoptera littoralis* (Boisd. 1833) (Lepidoptera, Noctuidae) (supplied as egg clutches by Syngenta, Switzerland) were reared in Petri dishes at 22–24 °C with a day/night 14–16 h photophase. An artificial diet consisting of 300 g·L^−1^ agar, 250 g·L^−1^ bean flour, 4.5 g ascorbic acid, 4.5 g ethyl *p*-hydroxybenzoate, 2.5 g vitamin E (all supplied by Sigma, Milan, Italy) dissolved in 17 mL of seed oil and 2 mL formaldehyde was used to feed larvae. Small cubes of the diet were placed into rearing dishes on pieces of aluminum foil. Regurgitation was enhanced by squeezing the larva with forceps behind the head. OS was then collected and stored at −20 °C until analysis.

For Vm analyses, plant responses were induced by esDNA, etDNA, and *S. littoralis* OS in leaves mechanically damage with forceps. For microscopic studies, mechanical damage was simulated using a pattern wheel. As negative controls, undamaged leaves were used. In order to compare the effect of esDNA with the action of the etDNA, leaf and larval homogenates and OS, we defined the timing of wounding at 30 min. That is, application was performed continuously for 30 min, while mechanical damage was performed once.

Pure homogenates from plants and larvae were obtained by grinding 1 g of fresh materials in a Tenbroeck glass tissue grinder with a Polytetrafluoroethylene (PTFE) pestle in the presence of MES buffer (1:10 ratio). The homogenate was then centrifuged at 5000× *g* for 30 min and 5% supernatant was used for Vm tests.

### 4.2. DNA Extraction and Sonication

Leaves of Lima bean and maize were collected and dried in oven at 60 °C for 72 h. For each DNA extraction, 800 mg of dried material was ground to powder in liquid nitrogen with mortar and pestle. Total DNA was isolated using both CTAB (CetylTrimethylAmmonium bromide) method, according to the Wilke’s protocol [41] and a DNeasy Plant Mini Kit as described by the manufacturer (Qiagen, Valencia, CA, USA, http://www.qiagen.com/).

*S. littoralis* III instar larvae (1 g) were lyophilized in liquid nitrogen and DNA was immediately extracted by CTAB method [41,42] and DNeasy Blood and Tissue Kit following manufacturer’s instructions (Qiagen, Valencia, CA, USA). Same methods were used to extract DNA from *S. littoralis* oral secretions. Yields and quality of DNA extraction were higher by using CTAB methods, which were therefore chosen to obtain pure DNA material throughout the experiment. Briefly, PVPP (polyvinylpolypyrrolidone, Sigma, Milan, Italy) powder was added to tissue before grinding only for DNA plant extraction. Tissues were homogenized with 10 mL of extraction buffer (100 mM Tris-HCl, pH 8.0, 1.4 M NaCl, 0.02 mM EDTA, 2% CTAB, and 0.2% β-mercaptoethanol). Afterward, and only for DNA extraction from *S. littoralis*, 150 μL of proteinase K (200 μg·mL^−1^) were added to samples and the homogenate was incubated at 65 °C for 2 h (1 h in the case of plant tissues). After centrifugation at 13,000 rpm for 10 min, an equal volume of chloroform:isoamyl alcohol (24:1) was added and samples were centrifuged at 13,000 rpm for 20 min. This step was repeated once and, after incubation for 30 min with a 1:100 volume of RNAse, the DNA was precipitated with isopropanol. Then, samples were centrifuged at 13,000 rpm for 10 min and the DNA pellet was washed twice with 76% aqueous ethanol, 0.2 M sodium acetate, and 70% aqueous ethanol subsequently. Finally, the pellet was air-dried and resuspended in PE buffer (5 mM Tris/HCl, pH 8.5).

DNA from leaves and *S. littoralis* larvae and OS were fragmented by sonication. This was performed with a Bandelin Sonopulse HD2070 (Bandelin, Berlin, Germany) at 90% power with a 1 s pulse for 30 min. Quality and length of sonicated band sizes were assessed by capillary gel electrophoresis using the Agilent 2100 Bioanalyzer (Agilent Technologies, Santa Clara, CA, USA) according to manufacturer’s instructions.

All DNA extracts were spectrophotometrically quantified at 260 nm on a NanoDrop ND 1000 Spectrophotometer (Thermo Scientific, Wilmington, DE, USA) and visually verified on 1.2% agarose gel using Gel Doc EZ System (Bio-rad, Hercules, CA, USA).

### 4.3. Membrane Potentials

Membrane potentials were determined in leaf segments. Glass micropipettes with a tip resistance of 4–10 MΩ and filled with 3 M KCl were used to measure the transmembrane potential (Vm). A Narishighe PE-21 puller (Narishige Scientific Instrument, Tokyo, Japan) was use to forge micropipettes used as micro-salt bridges to Ag/AgCl electrodes obtained. These micropipettes were inserted vertically in the tissue by means of a micromanipulator (for details see also [10]). Leaf segments were equilibrated for 60–120 min in 5 mM MES-NaOH (pH 6.0). A multichannel Ismatec Reglo (Ismatec SA, Glattbrugg, Switzerland) peristaltic pump (flow rate 1 mL·min^−1^) was used to perfuse the buffer. Topographical and temporal determination of Vm were initially performed to assess the electrode position, which was inserted between 0.5 and 1.5 mm from the leaf edge zone, where usually a significant Vm depolarization occurs after herbivory [22]. Vm variations were recorded through a digital port of a PC using a data logger. esDNA was assayed from 2 to 200 µg·mL^−1^ in both Lima bean and maize. Two hundred micrograms per milliliter of esDNA and etDNA were then assayed in both species, and 5% homogenate solutions were used. Five microliters of OS were used, according to previous protocols [43].

### 4.4. Determination of Intracellular Calcium Variations Using Confocal Laser Scanning Microscopy (CLSM) and Calcium Orange

Calcium orange dye (stock solution in DMSO, Molecular Probes, Leiden, The Netherlands) was diluted in 5 mM MES-Na buffer (pH 6.0) to a final concentration of 5 µM. This solution was applied on Lima bean and maize leaves attached to the plant as previously reported [22,43]. Incubation with calcium orange was performed for 1 h, then the leaf was mounted on a Leica TCS SP2 (Leica Microsystems Srl, Milan, Italy) multiband confocal laser scanning microscope (CLSM) stage. In order to assess the basic fluorescence levels as a control, the leaf was not separated from the plant. We used an average number of 8 leaves from several plants. Then 50 µL of 200 µg·mL^−1^ of either esDNA or etDNA were applied and after 30 min the calcium signature was observed. The microscope operates with a Krypton/Argon laser at 543 nm wavelength which excites calcium orange, resulting in green fluorescence, and at 568 nm wavelength mainly exciting chlorophyll, resulting in red fluorescence. All images were obtained with an objective HCX APO 40× in water immersion with an NA of 0.8. Scan speed was set at 400. The microscope pinhole was 0.064 mm and the average size depth of images was between 65 and 70 µm; the average number of sections per image was 25 and the final images were obtained by average analysis of Z-stacks. Image format was 1024 × 1024 pixels, 8 bits per sample and 1 sample per pixel. Images generated by the FluoView software were analyzed using the NIH image software as described earlier [44]. Briefly, the calcium fluorescence of several images was thresholded and analyzed by image analysis. For each plant species, the quantitative data were statistically processed and the highest value was compared to 100%. For calcium quantification, several zones of the leaf were covered in order to achieve the calcium local and systemic signaling without causing leaf damages due to the laser intensity. All other data were then recalculated in order to obtain the relative percentage of calcium signaling (see also [43]).

### 4.5. Capillary Electrophoresis and Cation Quantification

Lima bean, maize, and *S. littoralis* homogenates, as well as MES and PE buffers, were diluted 1:10 in Milli-Q Water and vortexed. Samples were filtered through a 0.45 µm cellulose filter and poured into polypropylene capillary electrophoresis (CE) injection vials. All CE experiments were carried out in triplicate by using an Agilent G1600 Capillary Electrophoresis System equipped with a diode array detector. The Cation Solution Kit (Agilent Technologies, Santa Clara, CA, USA) combined with a 64 cm length bare-fused silica capillary column (56 cm to detector window with a 50 µm internal diameter; Agilent Technologies, Santa Clara, CA, USA) was used for inorganic cation determination. Separation was performed at 25 °C at a 30 kV voltage. CE analyses were carried out with indirect UV detection at 310 nm with a bandwidth of 20 nm (reference 215 nm with a bandwidth of 10 nm). Pressure injections from sample vial was 50 mBar for 10 s followed by injection at 50 mBar for 2 s. Corresponding peaks identified in electropherograms were quantified by interpolation with a standard curve generated by using a reference Cation standard solution (Agilent Technologies, Santa Clara, CA, USA).

### 4.6. Statistical Analyses

A stem-and-leaf function of Systat Software 10 (Systat Software Inc., San Jose, CA, USA) was used to treat Vm measurement data to calculate the lower and upper hinge from the Gaussian distribution of values. The data were then filtered and the mean value was calculated along with the SE. At least five samples per treatment group were used for the statistical analysis of all other experimental data. Overall variation in the abundance of various cations were assessed on log-transformed data using the analysis of variance (ANOVA) while post hoc was used to test pairwise differences. Data are expressed as mean values ± standard error. To compare calcium images between control and treatment groups, analysis of variance (ANOVA) and Tukey’s test were performed.

## 5. Conclusions

The results of this work confirm that dicot plants such as Lima bean and monocot plants such as maize are able to respond to fragmented, but not integral, extracellular self-DNA. This response is specific and triggers early signaling events such as Vm depolarization and calcium signaling. Moreover, neither integral nor fragmented extracellular non-self-DNA have effects on these species, indicating a highly specific response. The rapidity of plant responses to esDNA is in favor of a direct system of perception (receptor?) rather than a longer-term interaction of esDNA with transcription and enzymatic activities. However, we cannot exclude the co-existence of both biological processes. Many questions remain open and further studies are required to better assess (1) the tissue and cell specificity of esDNA perception, since almost the same pattern of DNA fragmentation produces different responses; (2) its direct and indirect role in triggering cascades of events; (3) eventually leading to gene expression and post-translational modifications.

## Figures and Tables

**Table 1:** Capillary electrophoresis analysis of different cations present in extraction buffers, leaf and *Spodoptera littoralis* (*S.l.*) homogenates, and fragmented DNA at 200 µg·mL^−1^ concentration. Values are expressed as mg·L^−1^ (standard deviation). F subscripts are number of cases and degree of freedom, respectively.

Specifications	Cations				
	Ammonium	Potassium	Sodium	Calcium	Magnesium
MES buffer	6.31 (0.91)	6.15 (0.44)	39.48 (1.28)	28.01 (0.16)	3.08 (0.44)
PE buffer	4.84 (1.45)	16.33 (0.15)	2.71 (0.43)	1.57 (0.08)	2.71 (0.18)
Maize homogenate	3.41 (0.03)	1558.28 (21.92)	58.26 (4.01)	112.01 (4.08)	69.63 (4.80)
Lima bean homogenate	1.49 (0.39)	4307.91 (1.86)	26.21 (0.99)	516.21 (10.19)	208.60 (1.43)
*S.l.* homogenate	77.34 (9.54)	3872.34 (291.17)	554.43 (45.07)	53.68 (4.88)	462.29 (32.73)
Maize sonicated DNA	4.88 (0.22)	15.71 (0.14)	47.28 (1.39)	2.83 (0.19)	2.52 (0.04)
Lima bean sonicated DNA	4.86 (0.30)	15.86 (0.79)	124.28 (1.26)	3.39 (0.27)	2.28 (0.13)
Anova	F_17,6_ = 86.415	F_17,6_ = 11351.986	F_20,6_ = 1536.737	F_19,6_ = 3758.449	F_18,6_ = 1224.789
*p* values	*p* < 0.001	*p* < 0.001	*p* < 0.001	*p* < 0.001	*p* < 0.001
